# Managing a mavacamten non-responder in obstructive hypertrophic cardiomyopathy: A case report

**DOI:** 10.1016/j.jccase.2026.06.011

**Published:** 2026-07-09

**Authors:** Smita Scholtz, Felix Langkamp, Volker Rudolph, Jan Michael Federspiel, Vasco Sequeira

**Affiliations:** a Ruhr-University Bochum, Heart- and Diabetescenter NRW, Department of General and Interventional Cardiology/Angiology, Bad Oeynhausen, Germany; b Saarland University, Faculty of Medicine, Institute of Legal Medicine, Homburg (Saar), Germany; c Comprehensive Heart Failure Center (CHFC), University Clinic Würzburg, Würzburg, Germany

**Keywords:** Hypertrophic cardiomyopathy, Obstructive hypertrophic cardiomyopathy, Mavacamten, Non responder, Mitral valve disease

## Abstract

We report the case of a woman in her 40s with obstructive hypertrophic cardiomyopathy who remained symptomatic despite optimal medical therapy. A prior attempt of alcohol septal ablation was not performed due to unsuitable septal branch anatomy. Echocardiography demonstrated severe left ventricular outflow tract (LVOT) obstruction, systolic anterior motion (SAM) of the mitral valve (MV), and moderate-to-severe mitral regurgitation. Biomarkers indicated significant hemodynamic stress, and magnetic resonance imaging revealed myocardial fibrosis. Treatment with the cardiac myosin inhibitor mavacamten was initiated and despite stepwise escalation to 15 mg/day, LVOT gradients and symptoms persisted, classifying her as a treatment non-responder. Genetic testing detected a MYH7 variant of uncertain significance, potentially associated with reduced drug effectiveness. Given refractory obstruction, the multidisciplinary heart team recommended surgical septal myectomy. Intraoperative inspection of the mitral valve revealed not only SAM-related mitral regurgitation, but degenerative MV pathology requiring MV replacement. Postoperatively, LVOT obstruction resolved, and the patient became asymptomatic.

**Learning objective:**

This case underscores the clinical importance of recognition of non-response to mavacamten therapy. It highlights the need for careful evaluation of concomitant mitral valve pathology, which may significantly contribute to persistent symptoms beside left ventricular outflow tract obstruction. In selected patients, timely consideration of surgical intervention remains essential to achieve optimal hemodynamic improvement and symptomatic relief when pharmacological treatment alone is insufficient.

## Introduction

Obstruction of the left ventricular outflow tract (LVOT) strongly influences symptoms and outcome in patients with obstructive hypertrophic cardiomyopathy (oHCM). According to current European guidelines [1], [2], non-vasodilating β-blockers or non-dihydropyridine calcium channel blockers are the first choice of medical therapy. If symptoms persist mavacamten is recommended for symptom management and reduction of LVOT obstruction. Although septal-reduction therapies [alcohol septal ablation (ASA) or septal myectomy] have a level I evidence, they are only considered third-line options [2].

We present a case with oHCM who did not respond to mavacamten therapy, was successfully treated by surgical myectomy and mitral valve replacement, and discuss potential mechanisms of mavacamten-non-response.

## Case report

A woman in her 40s with oHCM presented with refractory heart failure symptoms [New York Heart Association (NYHA) class II] despite optimal medical therapy (β-blocker and the sodium-glucose co-transporter 2 inhibitor dapaglifozin) and prior attempt of ASA two years previously, which had not been performed due to unsuitable septal branch anatomy. Coronary angiography revealed a septal branch from the mid-left anterior descending artery (LAD) and a small branch from the ramus intermedius (RIM). In contrast echocardiography occlusion of the mid-LAD branch showed contrast opacification of the mid-septum rather than the basal septum. No proximal LAD septal branch was present. Occlusion of the RIM branch demonstrated extensive opacification of the septum and lateral wall, indicating an unsuitable target. Transthoracic echocardiography revealed a persistently thickened interventricular septum (maximal wall thickness in basal interventricular septum 24 mm, posterior wall thickness 22 mm), supranormal left ventricular (LV) ejection fraction (LVEF, 71%), systolic anterior motion (SAM) of the mitral valve (MV), moderate-to-severe regurgitation (vena contracta 6 mm, effective regurgitant orifice area 0,4 cm^2^), and a resting LVOT gradient of 180 mmHg (Video 1, Fig. 1). Cardiac magnetic resonance imaging (MRI) indicated myocardial fibrosis (late gadolinium enhancement). N-terminal pro B-type natriuretic peptide (NT-proBNP) was markedly elevated at 5520 pg/mL (normal <274 pg/mL), and high-sensitivity troponin I was 9 pg/mL (normal ≤20 pg/mL). Cardiopulmonary exercise testing revealed severely reduced exercise capacity, with a peak oxygen uptake of 15.6 mL/kg/min (normal >30 mL/kg/min for women in their 40s). Genetic testing identified a variant of uncertain significance in the *MYH7* gene (β-myosin heavy chain; c.4423C > Tp.Arg1475Cys). Given her persistent symptoms and obstruction, mavacamten (cardiac myosin inhibitor) was initiated at 5 mg/day after confirming non-poor CYP2C19 metabolic status. After 12 weeks, LVEF remained >70%, and the resting LVOT gradient had decreased only to 140 mmHg, with no clinical improvement. The dose was increased to 10 mg/day for four weeks and then to 15 mg/day for an additional eight weeks. However, her LVOT gradient remained 130 mmHg, LVEF was unchanged, NT-proBNP declined only to 3080 pg/mL, and symptoms persisted.

Given the persistent symptoms and insufficient hemodynamic improvement despite optimized medical therapy, further management options were carefully considered in the interdisciplinary heart team. Escalation of mavacamten beyond the recommended maximum dose, repetition of ASA, or discontinuation of mavacamten with continuation of β-blocker therapy alone were deemed inappropriate. In light of the ongoing severe LVOT obstruction, the patient was ultimately referred for septal myectomy, which was considered the most appropriate therapeutic strategy. Intraoperative assessment of the mitral valve demonstrated that, in addition to SAM–related mitral regurgitation, there was concomitant degenerative MV disease necessitating valve replacement.

**Fig. 1:**
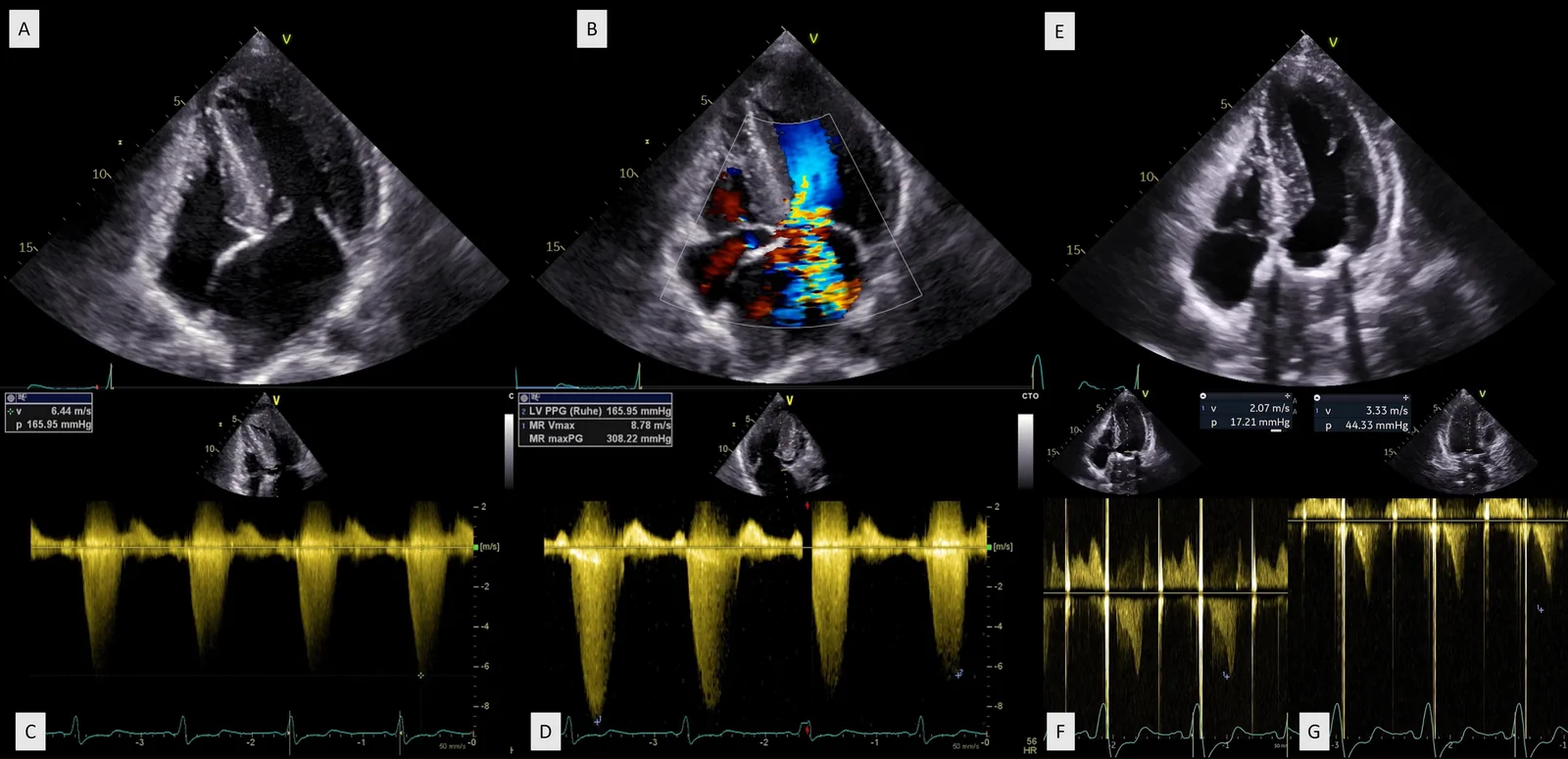
Transthoracic echocardiography of the patient with obstructive hypertrophic cardiomyopathy demonstrating massive obstruction of the left ventricular outflow tract at baseline and three months after septal myectomy and mitral valve replacement. (A) 4-chamber view with septal wall thickness of 24 mm. (B) Color Doppler with moderate mitral valve insufficiency (C) CW-Doppler at rest with peak LVOT gradient of 166 mmHg. (D) CW-Doppler at rest of the mitral valve insufficiency (first two beats) and LVOT gradient (last two beats). (E) 4-Chamber view with mechanical mitral valve prothesis. (F) CW-Doppler at rest with peak LVOT gradient of 17 mmHg (G) CW-Doppler under Valsalva manoeuvre with LVOT gradient of 44 mmHg. CW, continuous wave; LVOT, Left ventricular outflow tract.

## Discussion

Initially, oHCM is managed with negative inotropes (β-blockers, verapamil, disopyramide) to reduce hypercontractility [1], [2]. If symptoms or LVOT gradients remain severe (typically ≥50 mmHg at rest or with provocation) despite optimal medical therapy, septal reduction therapy is indicated [1], [2]. Surgery is generally preferred in younger patients or those with markedly thickened septa, whereas ASA may be considered in cases with suitable anatomy or elevated surgical risk. In the present patient with prior ASA treatment that had no lasting benefit, further conservative management was unlikely to suffice.

Mavacamten offers a targeted molecular approach by reducing sarcomere-level hypercontractility [3]. In the EXPLORER-HCM trial, it significantly lowered LVOT gradients and improved symptoms, although only ~27% achieved a complete response (defined as LVOT gradient <30 mmHg and NYHA class I) [4]. In the VALOR-HCM trial, 82% of patients treated with mavacamten became ineligible for septal reduction therapy after 16 weeks, compared with 23% on placebo [5]. Nonetheless, a notable subset (22/56 patients) had persistent gradients >50 mmHg [5]. There are multiple mechanisms contributing to LVOT obstruction in patients with oHCM, encompassing both functional and structural components. Functional mechanisms primarily involve dynamic factors such as SAM of the MV and altered ventricular loading conditions, whereas structural contributors include anatomical abnormalities such as basal septal hypertrophy, accessory or aberrant muscle bundles, and MV apparatus alterations. Importantly, the relative contribution and interplay of these mechanisms can vary considerably between patients. This heterogeneity in the underlying pathophysiology of LVOT obstruction is likely to influence the therapeutic response to mavacamten. Our patient showed a similar pattern, with minimal hemodynamic and symptomatic improvement despite high-dose therapy. This issue of mavacamten non-response, however, has been largely overlooked amid enthusiasm for this new therapy but is increasingly recognized in real-world settings: observational data suggest that patients with pathogenic sarcomere mutations (genotype-positive) respond more favorably than those without identified mutations (genotype-negative) [6]. Our patient carried a variant of uncertain significance, which may align with her non- response. Mechanistically, this may reflect how mavacamten interacts with the variant's location: while it binds to the myosin head region, its efficacy depends on the cooperative ability of the entire myosin dimer to adopt a folded-back, autoinhibited (“off”) state, an arrangement of the two myosin heads against the tail (i.e. super-relaxed myosin state) [7]. The identified variant lies within the myosin tail, maintaining such a autoinhibited conformation. Therefore, a structural disruption in the tail – as in the present case - could weaken the folded structure, leaving fewer myosin heads sequestered for mavacamten and potentially blunting the drug's effect. Thus, stabilization of the super-relaxed state by mavacamten may be influenced by external factors such as diet (e.g. *trans*-fatty acids binding in the same region) and stressors (i.e. increased sarcomere load or adrenergic stimulation [8]) that could counteract the effects of mavacamten in patients with hemodynamic compromise. Furthermore, rapid or ultra-rapid metabolizers of mavacamten may require a longer treatment period or dose adjustment to achieve sufficiently high plasma concentrations for a therapeutic effect on LVOT obstruction. Due to increased metabolic clearance, drug exposure in these patients may be lower, potentially delaying or attenuating the clinical response. Consequently, an extended treatment duration may be necessary to adequately assess the efficacy of mavacamten in this subgroup. Also, the HCM disease state may influence therapeutic efficacy of mavacamten: marked hypertrophy and myocardial fibrosis (compare MRI and histology; Fig. 2A-C) likely limiting the effect of mavacamten.

**Fig. 2:**
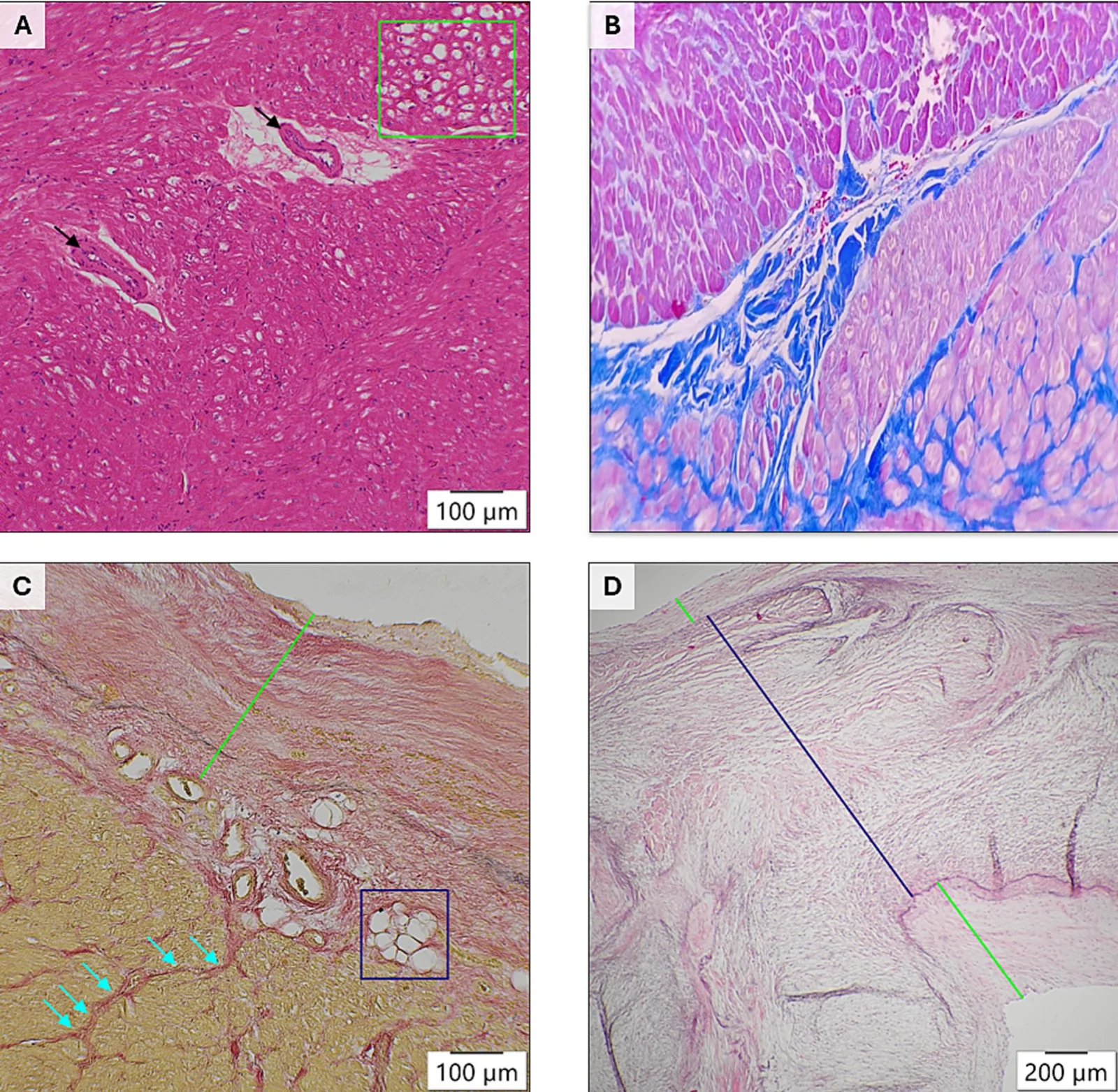
Histological assessment of the endomyocardial sample and the excised mitral valve. (A) Hematoxylin-eosin staining of the myocardium shows moderately enlarged cardiomyocytes and small, structurally unremarkable vessels. The blue rectangle exemplifies a patchy focal area with vacuolized cardiomyocytes. (B) Azocarmine-aniline blue (Azan) staining (100× magnification) highlights the moderately pronounced interstitial fibrosis appearing blue. Replacement fibrosis was not observed. (C) Elastica von Gieson staining. The bright blue arrows point towards areas affected by interstitial fibrosis (red staining). The green line marks an example of a thickened and fibrotic portion of the endocardial layer. Endocardial fibroelastosis was not observed. The dark blue rectangle highlights a group of fat cells localized in the transition zone from endocardium to myocardium. (D) Elastica hematoxylin-eosin staining of the mitral valve tissue. The spongiform layer (blue line) is markedly thickened compared to the surrounding connective tissue (green lines) indicating myxomatous degeneration of the mitral valve leaflets.

Repeat ASA was unlikely to succeed, given the absence of a suitable septal artery for effective alcohol delivery. In contrast, surgical myectomy directly removes obstructive tissue, immediately eliminates the gradient, and can treat concomitant MV disease. Correspondingly, the multidisciplinary heart team, incorporating the patient's preference, favored septal myectomy (excision of a 25 × 20 × 4 mm segment) with MV surgery (mechanical MV replacement; native valve degenerated and unsuitable for repair). Surgical inspection of the LVOT revealed severe narrowing with marked LV hypertrophy. Careful septal myectomy was performed and multiple tissue fragments were resected.

Her postoperative course was uncomplicated, she was discharged on postoperative day 8. Follow-up echocardiography showed complete resolution of resting LVOT obstruction (peak gradient 17 mmHg), with a mild mid-ventricular gradient on Valsalva (44 mmHg). The prosthetic MV functioned normally (Fig. 1). At three-month follow-up, she was asymptomatic (NYHA I).

As MV disease in HCM can negatively affect conservative treatment [9], the mitral regurgitation in the present case might have contributed to mavacamten non-response. That MV disease remained unchanged by mavacamten suggests a primary MV disease. The surgical and histological findings of severe MV degeneration (Fig. 2D) provide proof of the presence of primary MV disease complicating oHCM. The mitral regurgitation might also contribute to the unchanged LVEF as it is associated with LVEF overestimation [10].

## Conclusion

The presented case highlights (A) that a subset of patients with oHCM (i.e. uncertain/non-pathogenic genetic variants, primary MV disease) remains symptomatic despite mavacamten, (B) the potential impact of MV disease, (C) the need for septal reduction in, and (D) meticulous MV assessment (primary vs. secondary MV disease), and (E) the importance of interdisciplinarity for mavacamten non-responders.

## CRediT authorship contribution statement

SS, JMF, and VS were involved in study conception and design. Data collection and analysis were performed by SS, JMF, and VS. All authors were involved in manuscript writing. The final manuscript version was approved by all authors.

## Sources of funding

VS is supported by a research fund from 10.13039/100008021Bristol Myers Squibb and the 10.13039/501100001659Deutsche Forschungsgemeinschaft (DFG; project nos. 530849567 and 554784412).

## Additional contributions

We thank the patient for granting her written informed consent to publish the information presented. We gratefully acknowledge the Department of Pathology at Johannes Wesling University Hospital Ruhr University Bochum, Minden, Germany, for their essential contribution to the histological examinations.

## Declaration of competing interest

SS received travel expenses and speaker honoraria from Bristol Myers Squibb. VS has received research funding from Bristol Myers Squibb unrelated to this work. JMF, FL, and VR declare no conflict of interest.

## Appendix A Supplementary data

Video 1 Transthoracic echocardiographic sequences demonstrating the clinical course of a patient with hypertrophic obstructive cardiomyopathy (HOCM). Baseline examination revealed severe left ventricular outflow tract (LVOT) obstruction. Following treatment with mavacamten 15 mg daily, no significant reduction in LVOT obstruction was observed, indicating a lack of therapeutic response. After surgical septal myectomy, echocardiography demonstrated successful relief of LVOT obstruction with an excellent hemodynamic result.
